# Risk stratification and rapid geriatric screening in an emergency department – a quasi-randomised controlled trial

**DOI:** 10.1186/1471-2318-14-98

**Published:** 2014-08-30

**Authors:** Chik Loon Foo, Vivan Wing Yin Siu, Hou Ang, Madeline Wei Ling Phuah, Chee Kheong Ooi

**Affiliations:** 1 Emergency Department, Tan Tock Seng Hospital, 11 Jalan Tan Tock Seng, Singapore 308433, Singapore

**Keywords:** Geriatric screening, Elderly, Emergency department, Risk stratification

## Abstract

**Background:**

To determine if risk stratification followed by rapid geriatric screening in an emergency department (ED) reduced functional decline, ED reattendance and hospitalisation.

**Method:**

This was a quasi-randomised controlled trial. Patients were randomised by the last digit of their national registration identity card (NRIC). Odd number controls received standard ED care; even number patients received geriatric screening, followed by intervention and/or onward referrals. Patients were followed up for 12 months.

**Results:**

There were 500 and 280 patients in the control and intervention groups. The intervention group had higher Triage Risk Screening Tool (TRST) scores (34.3% vs 25.4% TRST ≥3, p = 0.01) and lower baseline Instrumental Activity of Daily Living (IADL) scores (22.84 vs 24.18, p < 0.01). 82.9% of the intervention group had unmet needs; 62.1% accepted our interventions. Common positive findings were fall risk (65.0%), vision (61.4%), and footwear (58.2%). 28.2% were referred to a geriatric clinic and 11.8% were admitted. 425 (85.0%) controls and 234 (83.6%) in the intervention group completed their follow-up. After adjusting for TRST and baseline IADL, the intervention group had significant preservation in function (Basic ADL -0.99 vs -0.24, p < 0.01; IADL -2.57 vs +0.45, p < 0.01) at 12 months. The reduction in ED reattendance (OR0.75, CI 0.55-1.03, p = 0.07) and hospitalization (OR0.77, CI0.57-1.04, p = 0.09) were not significant, however the real difference would have been wider as 21.2% of the control group received geriatric screening at the request of the ED doctor. A major limitation was that a large proportion of patients who were randomized to the intervention group either refused (18.8%) or left the ED before being approached (32.0%). These two groups were not followed up, and hence were excluded in our analysis.

**Conclusion:**

Risk stratification and focused geriatric screening in ED resulted in significant preservation of patients’ function at 12 months.

**Trial registration:**

National Healthcare Group (NHG) Domain Specific Review Board (DSRB) C/09/023. Registered 5th March 2009.

## Background

Emergency departments (EDs) across developed countries are seeing an 'epidemic’ of the elderly [1-5]. Unlike other epidemics, this threat was foreseen decades ago [6-8]. Despite its imminence, EDs remain unprepared for the challenges posed by a rapidly ageing population [9,10].

Elderly patients are different from their younger counterparts. Not only do they present with atypical features, multiple comorbidities, polypharmacy and cognitive impairment, they also harbor hidden needs not immediately visible in the initial assessment [1,10,11]. A patient with hypoglycemia may have acute (sepsis, change in medications) as well as chronic (depression, dysphagia) conditions that precipitated the low blood glucose. Simply reversing the hypoglycemia without identifying and addressing these unmet needs puts the patient at risk of another hypoglycemic episode. Delving deeper in order to unravel these clues require time, which unfortunately, is scarce in an overcrowded ED [12].

Elderly patients are a variegated group with differing risk profiles. Risk stratification helps to differentiate higher-risk from lower-risk seniors. Here, we used the Triage Risk Screening Tool (TRST) [13-15] to risk stratify, followed by rapid geriatric screening and intervention of at-risk seniors. Although both the Identification of Seniors at Risk (ISAR) [16,17] and TRST tools performed comparably in a previous studies [18,19] as well as in our local setting (unpublished pilot study), we chose the TRST because its lower 20% positive rate (vs ISAR’s 30%) was more manageable in our ED. Risk stratification was not performed on admitted patients. Such a two-stage workflow has been described before [13-17]; however, we believe this is the first time it has been tailored to the workflow of an extremely busy ED, and in an Asian setting.

## Method

### The setting

The setting was the ED of a 1,500-bedded acute care public hospital in Singapore, with an annual ED attendance of 160,000 patients. The eligibility criteria for inclusion in the study were: (1) patients aged 65 years old and above; (2) TRST score of 2 or more; and (3) patients who were planned for discharge. Patients were excluded if they were: (1) nursing home patients; (2) already on follow up with Geriatric Service; (3) of poor premorbid status i.e. bed-bound; (4) in advanced state of dementia, as defined by an inability to provide a reliable history. TRST score was performed by principal doctor of the patients prior to discharge.

### Study design

This was a quasi-randomised controlled trial. Patients were randomized using the last digit of their national registration identity card (NRIC) number. Odd-numbered patients were allocated to the control group; even-numbered patients were allocated to the intervention group. This method was used as it was easy to perform in a high ED traffic work environment. Recruitment was conducted only when the Geriatric Emergency Medicine (GEM) nurse was on duty i.e. 0900 hours to 1700 hours on weekdays; and 0800 hours to 1300 hours on Saturdays. Verbal consent was obtained after allocation: the control group via a telephone call by the research assistant (RA) after the patient had already left ED; intervention group by the GEM nurse whilst the patient was still in ED. The following data was collected at the point of recruitment: (1) Age; (2) Gender; (3) Race; (4) Patient Acuity Category (PAC) score; (5) TRST score; (6) baseline modified Barthel’s Index of Activities of Daily Living; BADL (out of 20); and (7) baseline Lawton’s Instrumental Activities of Daily Living score; IADL (out of 30). The latter two tools were used because they were validated for use over the telephone [20,21]. The function prior to the current injury or illness was solicited. The principal doctor of the patients was not blinded to the study.

### Control group

The control group patients received standard care. They were contacted via telephone by the research assistant (RA) within 3 days of discharge from ED. The purpose of the phone-call was to obtain consent for study participation, as well as baseline BADL and IADL scores.

### Intervention group

After consenting to the study, the intervention group patients were assessed by the GEM nurse while still in ED, prior to discharge. The nurse performed focused geriatric screening using a 15-question screening form. This was an abbreviated version of the screening tool used in our previous study [22]. The focused areas included cognition, mood, continence, visual acuity and hearing, mobility and social issues. Medication reconciliation and a postural blood pressure were also performed. Each assessment lasted between 15–30 minutes. Clinically significant findings were addressed immediately where possible. Referrals to allied health professionals e.g. physiotherapist and occupational therapist were done as deemed necessary. When appropriate, patients were referred to the geriatric assessment clinic, post acute care at home (PACH), transitional services and community outreach services. Upon discharge, education and advice regarding fluid management, falls prevention, sleep hygiene and active lifestyle were provided where necessary.

### Outcome measures and follow up duration

The primary outcome of the study was a change in the patient’s functional status, measured by BADL and IADL scores. The secondary outcomes were healthcare utilization, as measured by ED reattendance and rehospitalisation. The patients were followed up via telephone call at 3, 6, 9 and 12 months to ascertain their BADL and IADL scores. Subsequent ED attendance and hospitalization were obtained via the national electronic medical records.

### Statistical analysis

The sample size was calculated based on a previous study done in department’s short stay ward [22]. It was decided that a change of score of 2 or more in the BADL and ADL was considered as clinically important. To ensure the study had at least 80% power with a two-sided level α of 0.05 to detect a change of BADL or ADL score of 1 between the control and intervention group, a sample size of 143 patients was required in each arm. When analysed, continuous data was compared using student’s *t*-test and Mann–Whitney *U* test where applicable. Categorical data was analysed using Pearson’s chi-square test. Logistic regression analyses were performed for the secondary outcomes of ED reattendance and rehospitalisation. The data was analysed with an intention-to-treat (ITT) principle. Data analysis was done using SPSS (version 19; SPSS, Inc., Chicago, IL).

### Ethics approval

The study was supported by the Singapore Ministry of Health (MOH)’s Healthcare Quality Improvement and Innovation Fund (HQI2F). The Domain Specific Review Board of National Healthcare Group approved the study.

## Results

### Recruitment

**Figure 1:**
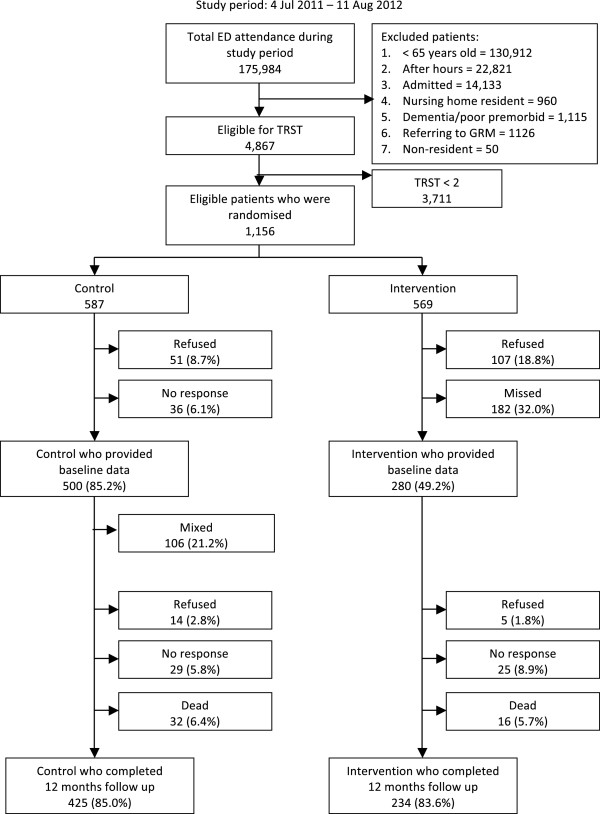
Recruitment flow diagram.

Between 4th July 2011 and 11th August 2012, 1156 patients were eligible to be included in the study (Refer Figure 1). After randomization, there were 587 patients in the control group and 569 patients in the intervention group. In the control group, 51 patients (8.7%) refused participation and 36 patients (6.1%) did not respond to telephone calls, leaving 500 patients who provided baseline data. In the intervention group, 107 patients (18.8%) refused participation and 182 patients (32%) were not screened by GEM nurse for various reasons, for example the patient was unable to wait for screening, or the GEM nurse was occupied with another case (categorized as the 'Missed’ group), leaving 280 patients who provided baseline data. The 'Missed’ group was excluded from the analysis as there was a high degree of missing data. Within the control group, 106 patients (21.2%) were referred to the GEM nurse for screening at the discretion of the principal doctor. This group was labelled as the 'Mixed’ group, and was analysed as part of the control group according to the intention-to-treat principle. 425 patients (85%) and 234 patients (83.6%) from the control and intervention group respectively completed follow up at 12 months.

### Baseline

The baseline age, gender, racial distribution and patient acuity category (PAC) were similar in both groups (refer Table 1). However, there was higher proportion of patients with a TRST score between 3–6 in the intervention group (34.3% vs. 25.4%, p = 0.01) and the difference was statistically significant. Similarly, there was a statistically significant difference in the baseline mean IADL scores between the groups, the intervention group being more dependent (22.84 vs. 24.18, p < 0.01). This suggested that the patients in the intervention group were more frail.

**Table 1:** Patient characteristics and demographics, based on different groupings and subgroupings

	***n***	**Control + Mixed**		**Intervention only**			**Control only**		**Mixed only**		**Intervention + Mixed**		**Missed**	
		**500**		**280**			**394**		**106**		**386**		**182**	
Age	Median	77		77		*p* = 0.21	77		78		78		77	
Gender	M	219	43.8%	130	46.4%	*p* = 0.53	184	46.7%	35	33.0%	165	42.7%	80	44.0%
	F	281	56.2%	150	53.6%		210	53.3%	71	67.0%	221	57.3%	102	56.0%
Race	Chinese	415	83.0%	236	84.3%	*p* = 0.76	327	83.0%	88	83.0%	324	83.9%	145	79.7%
	Malay	30	6.0%	18	6.4%		24	6.1%	6	5.7%	24	6.2%	19	10.4%
	Indian	48	9.6%	21	7.5%		38	9.6%	10	9.4%	31	8.0%	15	8.2%
	Others	7	1.4%	5	1.8%		5	1.3%	2	1.9%	7	1.8%	3	1.6%
PAC Score	1	5	1.0%	0	0.0%	*p* = 0.32	4	1.0%	1	0.9%	1	0.3%	6	3.3%
	2	268	53.6%	155	55.4%		210	53.3%	58	54.7%	213	55.2%	100	54.9%
	3	226	45.2%	125	44.6%		180	45.7%	46	43.4%	171	44.3%	75	41.2%
	4	1	0.2%	0	0.0%		0	0.0%	1	0.9%	1	0.3%	1	0.5%
TRST Score	2	373	74.6%	184	65.7%	*p* = 0.01	308	78.2%	65	61.3%	249	64.5%	129	70.9%
	3	102	20.4%	73	26.1%		71	18.0%	31	29.2%	104	26.9%	48	26.4%
	4	22	4.4%	21	7.5%		13	3.3%	9	8.5%	30	7.8%	4	2.2%
	5	2	0.4%	2	0.7%		2	0.5%	0	0.0%	2	0.5%	1	0.5%
	6	1	0.2%	0	0.0%		0	0.0%	1	0.9%	1	0.3%	0	0.0%
	≥3	127	25.4%	96	34.3%		86	21.8%	41	38.7%	137	35.5%	53	29.1%
0BADL	Mean	18.67		18.45		*p* = 0.27	18.64		18.77		18.54		NA	
0IADL	Mean	24.18		22.84		*p* < 0.01	24.37		23.47		23.02		NA	

### Positive findings & intervention

**Figure 2:**
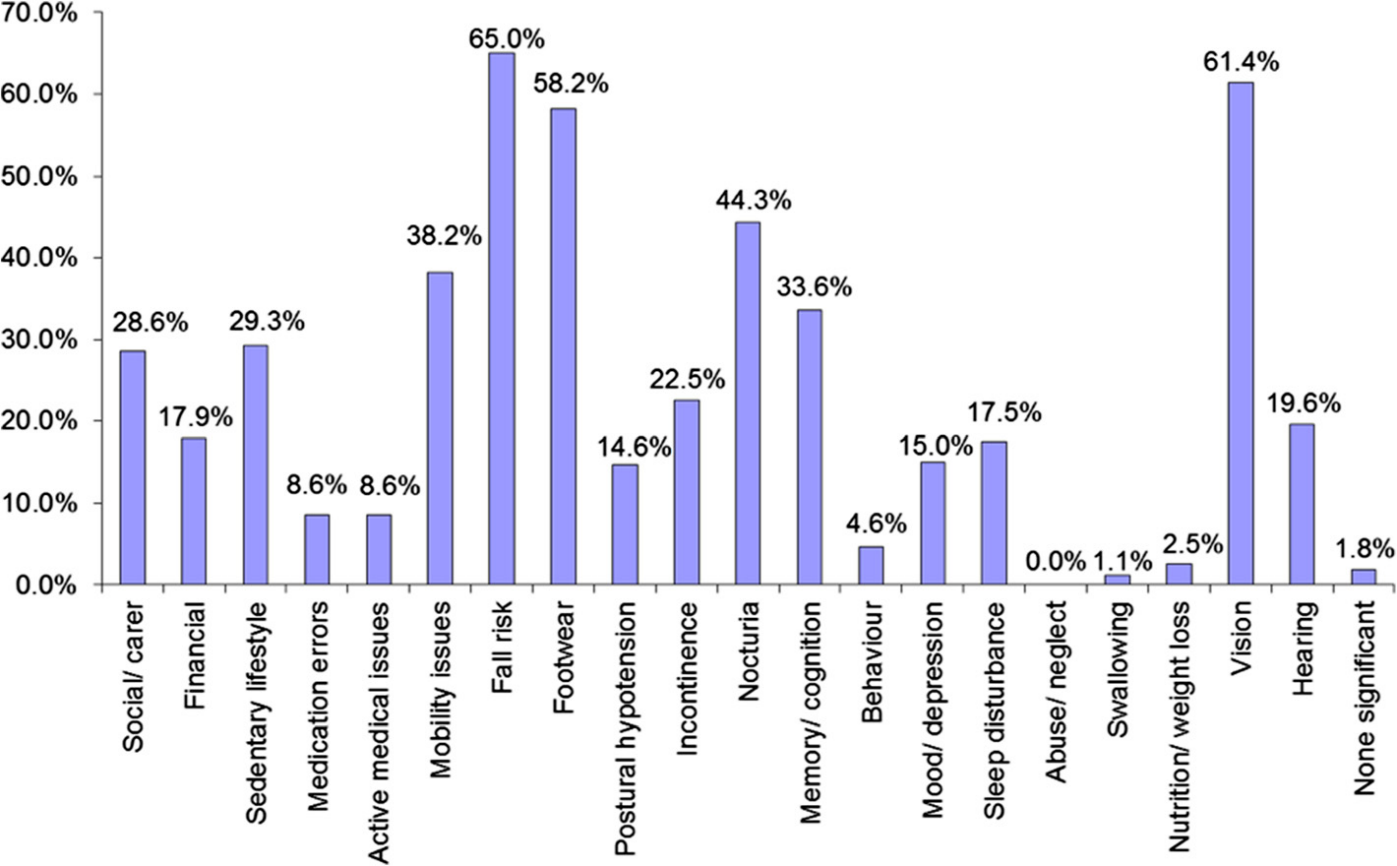
Positive findings during rapid geriatric screening.

**Figure 3:**
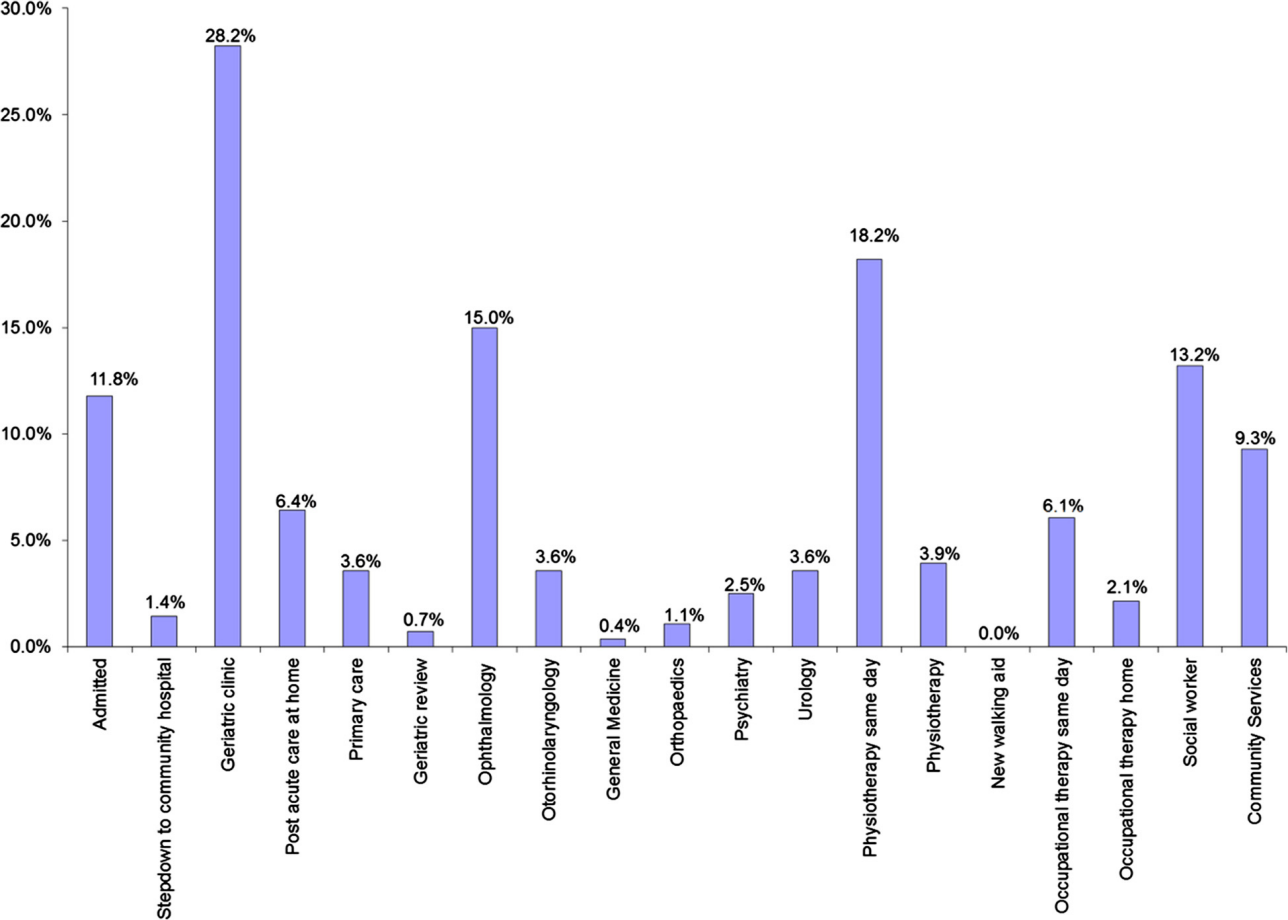
Interventions following rapid geriatric screening.

Within the intervention group, 65.0% of patients were found to have significant fall risk, 61.4% had visual impairment, and 58.2% had improper footwear (Figure 2). 82.9% required some form of geriatric intervention; however, 20.7% declined our suggestions. Amongst the interventions, 18.2% were referred to a physiotherapist on the same day, 28.2% to the geriatric clinic for geriatric syndrome(s) and 15.0% to the ophthalmologist for visual disturbance. PACH medical service was arranged for 6.4% for subsequent home visits. Finally, 11.8% of patients were, through geriatric screening, found to be sicker than expected and were admitted to the ward as a for further management (Figure 3).

### Outcome measures

**Figure 4:**
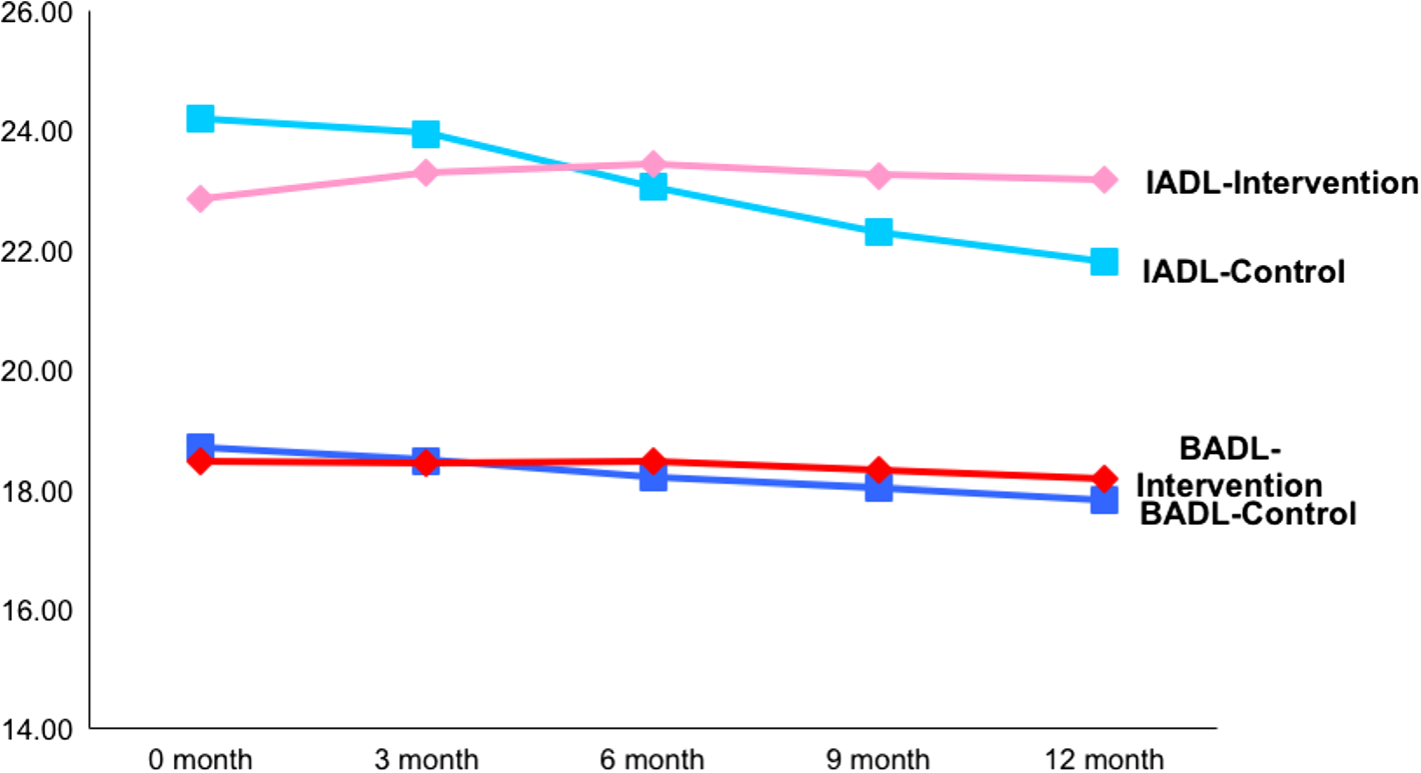
Graphical representation of basic (BADL) and Instrumental (IADL) activities of daily living scores over time.

Compared to the intervention group, BADL and IADL scores of patients in the control group appeared to have deteriorated, and the difference was statistically significant starting at 3 months. BADL scores for both control and intervention groups deteriorated over 12 months, but the degree of deterioration for the control group was more (-0.99 vs. -0.24, p < 0.01). Whilst the IADL scores for the control group also deteriorated over 12 month, the scores for patients in the intervention group actually improved, and the difference was statistically significant (0.45 vs. -2.57, p < 0.01; Table 2 and Figure 4).

**Table 2:** Bivariate analysis of ED reattendance, hospitalisation and basic (BADL) & instrumental (IADL) activities of daily living

	**Control**		**Intervention**		
	***n***	**%**	***n***	**%**	
ED reattendance at 3 months	186	37.2%	103	36.8%	*p* = 0.97
Hospitalisation at 3 months	144	28.8%	78	27.9%	*p* = 0.84
ED reattendance at 6 months	254	50.8%	134	47.9%	*p* = 0.48
Hospitalisation at 6 months	202	40.4%	107	38.2%	*p* = 0.60
ED reattendance at 9 months	299	59.8%	153	54.6%	*p* = 0.19
Hospitalisation at 9 months	241	48.2%	123	43.9%	*p* = 0.28
ED reattendance at 12 months	330	66.0%	171	61.1%	*p* = 0.19
Hospitalisation at 12 months	269	53.8%	139	49.6%	*p* = 0.30
	*n*	difference	*n*	difference	
BADL at baseline	500	0.00	280	0.00	*p* = 0.10
IADL at baseline	500	0.00	280	0.00	*p* < 0.01
BADL difference at 3 months	479	-0.25	269	0.00	*p* < 0.01
IADL difference at 3 months	479	-0.33	269	0.53	*p* < 0.01
BADL difference at 6 months	469	-0.53	260	0.03	*p* < 0.01
IADL difference at 6 months	469	-1.24	260	0.60	*p* < 0.01
BADL difference at 9 months	439	-0.78	248	-0.08	*p* < 0.01
IADL difference at 9 months	439	-2.02	248	0.63	*p* < 0.01
BADL difference at 12 months	423	-0.99	234	-0.24	*p* < 0.01
IADL difference at 12 months	423	-2.57	234	0.45	*p* < 0.01

In terms of healthcare utilization, we did not find any statistical difference in the ED reattendance and hospital admissions between the control and intervention group. Even after adjusting for TRST scores and IADL scores (which were both statistically different at baseline), the odd ratios remain insignificant (Table 3). The number of deaths in both groups was similar (32; 6.4% vs 16; 5.7%).

**Table 3:** ED reattendance and hospitalisation rates, before and after adjusting for TRST and IADL scores (intention-to-treat analysis)

**ED attendance**				
**Follow up interval**	**Unadjusted OR**	**95% CI**	Adjusted OR*	95% CI*
3 months	0.98	0.73 to 1.33	0.91	0.67 to 1.24
6 months	0.89	0.66 to 1.19	0.82	0.61 to 1.11
9 months	0.81	0.60 to 1.09	0.74	0.55 to 1.01
12 months	0.81	0.60 to 1.09	0.75	0.55 to 1.03
**Hospitalisation**				
**Follow up interval**	**Unadjusted OR**	**95% CI**	Adjusted OR*	95% CI*
3 months	0.96	0.69 to 1.32	0.88	0.63 to 1.22
6 months	0.91	0.68 to 1.23	0.84	0.62 to 1.14
9 months	0.84	0.63 to 1.13	0.76	0.56 to 1.03
12 months	0.85	0.63 to 1.14	0.77	0.57 to 1.04

*Adjusted to TRST scores and IADL scores.

We also performed a per protocol (PP) analysis, combining the 'Mixed’ group with the intervention group, both groups having received geriatric screening. Here, results were even more positive, demonstrating reduction in ED reattendance at 6, 9 and 12 months after adjusting for TRST and IADL (Table 4). There was significant reduction in hospitalisation at only 9 months.

**Table 4:** ED reattendance and hospitalisation rates, before and after adjusting for TRST and IADL scores (per protocol analysis)

**ED reattendance**				
**Follow-up interval**	**Unadjusted OR**	**95% CI**	Adjusted OR*	95% CI*
3 months	0.90	0.67 to 1.20	0.81	0.60 to 1.09
6 months	0.77	0.58 to 1.01	0.69	0.52 to 0.92
9 months	0.68	0.51 to 0.90	0.60	0.45 to 0.81
12 months	0.77	0.57 to 1.03	0.71	0.52 to 0.96
**Hospitalisation**				
**Follow-up interval**	**Unadjusted OR**	**95% CI**	Adjusted OR*	95% CI*
3 months	0.93	0.68 to 1.27	0.83	0.61 to 1.15
6 months	0.86	0.65 to 1.15	0.78	0.58 to 1.05
9 months	0.76	0.58 to 1.01	0.68	0.51 to 0.91
12 months	0.85	0.64 to 1.13	0.77	0.57 to 1.03

*Adjusted to TRST scores and IADL scores.

## Discussion

Providing holistic geriatric care in a busy emergency department is new and challenging. Current consensus is that dedicated personnel is needed, preferably a multidisciplinary team. Risk stratification is necessary because not every patient would benefit from comprehensive assessment. There also needs to be greater integration with downstream stepdown and community services, more than what traditionally exists in most EDs [10,23-26].

Results of geriatric screening in the ED have been mixed. The first attempt was by Miller in 1996, which showed no benefit of comprehensive geriatric screening in the ED [27]. The Montreal team who created the ISAR tool found significant reduction in functional decline at 4 months at no higher costs, but also showed a paradoxical increase in ED reattendance [16,17]. The Cleveland group who invented the TRST score found no change in ED reattendance or hospitalisations; there was only reduction in nursing home admissions at 30 and 120 days [13-15]. The DEED II study in Australia risk stratified solely by age (≥75 years old) and had variegated results: less functional decline at 6 but not at 18 months, and significant reduction in emergency admissions at 18 months. Most of these studies involved a geriatric nurse, often an advanced practitioner nurse (APN), and commonly extended geriatric assessment and intervention to the patients’ homes [13-17,27,28].

We described a fully ED-based risk stratification and geriatric screening service, conducted by rotating ED (non-APN) nurses trained in geriatric care. Healthcare settings without a strong home-care set-up and without a mature pool of APN’s would be interested in our findings. We tried to simulate real-life conditions rather than 'study conditions’, and our results have allowed us to continue this service exactly as it was during the study period. Risk stratification followed by rapid geriatric screening has become standard of care.

As with our previous experience, there was a higher number of refusals in the intervention group [22]. This is expected, as geriatric screening involves extending the patient’s ED length of stays, which is not often favored by patients or family members. We had initially postulated that these patients may have left ED because they were more 'well’; however, subgroup analysis of their age, PAC and TRST scores did not support this.

21.2% of patients in the control group received geriatric screening at the request of the ED principal doctor ('mixed’ group). This was allowed in order to replicate 'real-life’ conditions in the ED. These patients remained in the control group and their outcomes were analysed in line with intention to treat principle. Had the patient allocation been strict (i.e. no geriatric screening for control group patients), the true reduction in functional decline, ED reattendance and hospitalisation rates would be more pronounced.

Interestingly, 11.8% of intervention group patients were discovered – during screening – to have sufficiently high risk needs to warrant admission, with a subsequent median length-of-stay of 6 days. This has not been reported in other similar studies, and yet is consistent with our previous experience, where 10.2% of 24-hour short stay ward elders were turned-inpatient for further evaluation [8]. The 33 patients in this study might have potentially been unsafely discharged had geriatric screening not been performed. As a result, currently our ED doctors often use geriatric screening as a safety net when discharging a frail elderly.

Whilst ITT analysis did not elucidate any difference in ED reattendance and hospitalisation rates, per protocol analysis revealed a sustained reduction in ED reattendance over 6, 9 and 12 months. It is likely that the 'Mixed’ group was a subjectively more frail subset within the control group, thus prompting the ED doctor to request for geriatric screening. This is supported by more of them having higher TRST scores of ≥3 (38.7%) compared to patients in the control group who were un-screened (21.8%). Our per protocol results suggests that geriatric screening may be particularly beneficial to a frailer group of ED elders.

This study has demonstrated that geriatric screening of at-risk ED elders prior to discharge resulted in a consistent and sustained preservation of function over 12 months. In fact, IADL actually improved at 3, 6, 9 and 12 months compared to baseline, which goes against the natural history of an elderly’s function over time. We propose two primary reasons for this. Firstly, since our 2008 study describing geriatric screening in a 24-hour short stay ward [22], we have expanded our GEM practice. More geriatric services have been added in the ED, such as medication reconciliation, occupational therapist assessments, and geriatric review clinics. We have also established new collaborations with post-acute care at home (PACH), Virtual Hospital (who follows up on frequent attenders), and home help providers (for patients who require ADL support). These initiatives are consistent with some recent GEM recommendations [23,29], allowing the intervention group to be supported by a mature and robust system in their transition from ED to home.

Secondly, the vast majority of local patients do not have a regular general practitioner, and geriatric screening is not commonly performed at primary care. Majority of our patients would have heard the question 'do you feel sad?’ for the first time during their encounter with our GEM nurse. Many of the accompanying relatives were grateful for the extra time taken, and for the more holistic approach they experienced during the visit. It is possible that geriatric screening created a new awareness within the patient and family, prompting them to pay extra attention towards maintenance of the patient’s function. The education and discharge advices provided by the GEM nurse may have also empowered the patient to take more responsibility for their own health. Furthermore, the patient themselves would have suffered a minor injury or illness that prompted this ED visit. This vulnerable state possibly made them more receptive to the interventions we proposed.

### Limitations

There were several limitations in our study. In terms of study design, we opted for a quasi-randomised instead of a true randomized controlled trial. We made this decision in order to reduce disruption to the ED operations. However, we feel that our results were still valid as the baseline characteristics between the control and intervention groups were quite similar. Baseline mean TRST scores and IADL scores, which were the only variables that were statistically different at baseline, were adjusted in the logistic regression analysis. Our patient population was a convenience sample that matched the duty hours of the GEM nurses. This may have led to selection bias. This was a necessary compromise in this study.

The major shortcoming of our study was that a large proportion of patients who were randomized to the intervention group either refused (18.8%) or left the ED before being approached (32.0%). These two groups were not followed up, and hence were excluded in our analysis. The size of these groups would have impacted our study results, although it is uncertain in which direction.

Another significant limitation is that the RA who collected BADL and IADL scores via telephone call was not blinded to patients’ group allocation. Although observer bias maybe an issue, the fact that the BADL and IADL scoring checklists are objective would have reduced this to a minimum. Furthermore, ED reattendance and hospitalisation data were retrieved via electronic medical records and would not be subject to bias. Finally, we did not collect data regarding quality of life as well as patient satisfaction levels for GEM screening in ED.

## Conclusion

Risk stratification followed by focused geriatric screening is feasible and effective even in a busy ED. We have shown significant and sustained in preservation of function over 12 months. Multidisciplinary assessment as well as strong interdisciplinary collaboration are key components of an effective geriatric emergency service.

## Abbreviations

APN: Advanced practitioner nurse; BADL: Basic activity of daily living; ED: Emergency department; DEED: Discharged elderly from the emergency department; GEM: Geriatric emergency medicine; HQI2F: Healthcare quality improvement and innovation fund; IADL: Instrumental activity of daily living; ISAR: Identification of seniors at risk; MOH: Ministry of health; NRIC: National registration identity card; PAC: Patient acuity category; PACH: Post acute care at home; RA: Research assistant; TRST: Triage risk screening tool.

## Competing interests

This study was supported by the Ministry of Health’s Healthcare Quality Improvement and Innovation (HQI2) Fund 2011/11.

## Authors’ contributions

CLF, VWYS, MWLP and HA conceived the study, designed the trial, supervised the conduct of the trial and data collection, undertook recruitment of patients and managed the data, including quality control. CLF obtained research funding. CKO provided statistical advice on study design and analyzed the data. CLF and CKO drafted the manuscript, and all authors contributed substantially to its revision. CLF takes responsibility for the paper as a whole. All authors read and approved the final manuscript.

## Authors’ information

CLF leads the geriatric emergency medicine team in Tan Tock Seng Hospital, whose emergency department is the busiest in Singapore. Since 2006, his team has strived to deliver holistic, tailored and quality care to ED elders. VSWY, HA and MWLP are senior members of the GEM team. CKO is also an emergency consultant. He holds a Master of Science and is the research lead in the department.

## Pre-publication history

The pre-publication history for this paper can be accessed here:

http://www.biomedcentral.com/1471-2318/14/98/prepub
